# Characterization and Metabolism of Drug Products Containing the Cocaine-Like New Psychoactive Substances Indatraline and Troparil †

**DOI:** 10.3390/metabo14060342

**Published:** 2024-06-18

**Authors:** Sascha K. Manier, Paula Mumber, Josef Zapp, Niels Eckstein, Markus R. Meyer

**Affiliations:** 1 Department of Experimental and Clinical Toxicology and Pharmacology, Institute of Experimental and Clinical Pharmacology and Toxicology, Saarland University, Center for Molecular Signaling (PZMS), 66421 Homburg, Germany; 2 Applied Pharmacy, University of Applied Sciences Kaiserslautern, Campus Pirmasens, 66953 Pirmasens, Germany; 3 Department of Pharmaceutical Biology, Saarland University, 66123 Saarbrücken, Germany

**Keywords:** HPLC-HRMS/MS, new psychoactive substances, indatraline, troparil, metabolism

## Abstract

With a rising demand of cocaine over the last years, it is likely that unregulated new psychoactive substances with similar effects such as indatraline ((1*R*,3*S*)-3-(3,4-dichlorophenyl)-*N*-methyl-2,3-dihydro-1*H*-inden-1-amine) and troparil (Methyl (1*R*,2*S*,3*S*,5*S*)-8-methyl-3-phenyl-8-azabicyclo[3.2.1]octane-2-carboxylate) become popular as well. Both substances share a similar pharmacological profile as cocaine, while their potency is higher, and their duration of action is longer. This study investigated their metabolic fate in rat urine and incubations using pooled human liver S9 fraction (pHLS9). Indatraline formed two phase I and four phase II metabolites, with aromatic hydroxylation and glucuronidation being the main metabolic steps. All metabolites were detected in rat urine, while the parent compound was not detectable. Although low in abundance, indatraline metabolites were well identifiable due to their specific isotopic patterns caused by chlorine. Troparil formed four phase I and three phase II metabolites, with demethylation being the main metabolic step. Hydroxylation of the tropane ring, the phenyl ring, and combinations of these steps, as well as glucuronidation, were found. Phase I metabolites were detectable in rat urine and pHLS9, while phase II metabolites were only detectable in rat urine.

## 1. Introduction

**Figure 1:**
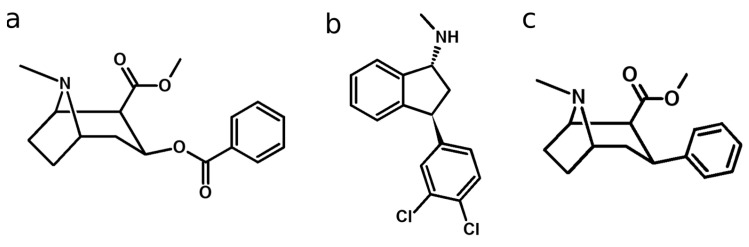
Chemical structures of cocaine (**a**), indatraline (**b**), and troparil (**c**).

Between 2015 and 2021 the amount of seized cocaine has roughly doubled, with the demand steadily increasing to an estimated 22 million cocaine users in 2021 [1,2]. However, the expansion of plant-based drugs of abuse is usually limited to harvesting and procession times [1]. It is likely that consumers and drug dealers might fall back to alternative compounds with similar pharmacology such as new psychoactive substances (NPSs). A similar behavior was reported with heroin in the context of the opioid crisis, where fentanyl analogs led to an increase in overdoses [3]. Two such NPSs with cocaine-like effects are indatraline ((1*R*,3*S*)-3-(3,4-dichlorophenyl)-*N*-methyl-2,3-dihydro-1*H*-inden-1-amine) and troparil (Methyl-(1*R*,2*S*,3*S*,5*S*)-8-methyl-3-phenyl-8-azabicyclo[3.2.1]octane-2-carboxylate). The chemical structures of these NPSs are depicted in Figure 1. Indatraline acts as an antidepressant that inhibits the reuptake of dopamine, serotonin, and noradrenaline [4]. It is described as producing cocaine-like effects, with a slower onset and a longer duration of action [5]. These properties led to the consideration of indatraline as a possible candidate for the treatment of cocaine dependence, although experiments in rhesus monkey hinted to side-effects such as mild anemia and weight-loss [5].

Troparil is a phenyltropane derivative that was first synthesized by Clarke et al. in 1970 [6]. It acts like indatraline as a reuptake inhibitor of dopamine, norepinephrine, and serotonin, producing cocaine-like effects [7,8]. Its ability to inhibit dopamine and noradrenaline is four- to five-times higher than that of cocaine, while inhibition of serotonin reuptake is lower [7]. Intoxications with either of the compounds were not described so far, but troparil was identified in samples collected from drug users in Warsaw, indicating its active consumption at least in Europe [9]. A reason why acute intoxication may have not been reported so far is the missing knowledge about detectable biomarkers in biological samples. In order to elucidate the cause of a patient’s condition, it is essential for clinical toxicologists to be able to confirm findings using knowledge about their metabolic patterns [10]. The determination of plasma concentrations is usually not necessary, since the mere detection of a psychoactive substance and its metabolites can provide a suitable explanation and thus enable physicians to treat patients properly, especially if it is a drug of abuse. Knowledge about their metabolism is therefore important for correct treatment of intoxicated patients and forensic case interpretation.

This is why this study aimed to investigate a troparil and indatraline drug product concerning their purity and adulteration, as well as the metabolic fate of the two compounds in rats and incubations using pooled human liver S9 fraction (pHLS9), to facilitate their detection in toxicological samples after assumed consumption. While the characterization shall be conducted using nuclear magnetic resonance (NMR), high-performance liquid chromatography coupled to high-resolution tandem mass spectrometry (HPLC-HRMS/MS) shall be used in metabolism studies.

## 2. Materials and Methods

### 2.1. Chemicals and Reagents

Indatraline and troparil were obtained by University of Applied Sciences Kaiserslautern from an online vendor of NPSs based in Germany. 3′-phosphoadenosine-5′-phosphosulfate (PAPS), *S*-(5′-adenosyl)-l-methionine (SAM), dithiothreitol (DTT), reduced glutathione (GSH), acetylcarnitine transferase (AcT), acetylcarnitine, acetyl coenzyme A (AcCoA), MgCl_2_, K_2_HPO_4_, KH_2_PO_4_, superoxide dismutase (SOD), isocitrate dehydrogenase, isocitrate, tris, ammonium formate, and formic acid were obtained from Sigma (Taufkirchen, Germany), and NADP-Na_2_, acetonitrile, and methanol were obtained from VWR (Darmstadt, Germany). Water was purified using a Millipore filtration unit. pHLS9 (20 mg protein/mL, from 30 individual donors), UGT reaction mix solution A (25 mM UDP-glucuronic acid), and UGT reaction mix solution B (250 mM Tris-HCl, 40 mM MgCl_2_, and 0.125 mg/mL alamethicin) were obtained from Corning (Amsterdam, The Netherlands). for qNMR was obtained from Honeywell Fluka (Morristown, NJ, USA).

### 2.2. In Vitro Incubations Using pHLS9

The pHLS9 incubations were carried out in accordance with previous publications [11,12]. The final incubation volume was 150 µL with a final protein concentration of 2 mg/mL. After preparation of the preincubation mixture consisting of 25 µg/mL alamethicin (UGT reaction mix solution B), 90 mM phosphate buffer (pH 7.4), 2.5 mM Mg^2+^, 2.5 mM isocitrate, 0.6 mM NADP^+^, 0.8 U/mL isocitrate dehydrogenase, 100 U/mL superoxide dismutase, 0.1 mM AcCoA, 2.3 mM acetyl carnitine, and 8 U/mL carnitine acetyltransferase, preincubation was performed at 37 °C and 200 rpm for 10 min. Subsequently, 2.5 mM UDP-glucuronic acid (UGT reaction mixture solution A), 40 µM aqueous PAPS, 1.2 mM SAM, 1 mM DTT, 10 mM GSH, and 25 µM substrate in phosphate buffer were added to the preincubation mixture. Reactions were started by adding 25 µM substrate. Incubations were conducted using a Cell Media TS pro (Zeitz, Germany) at 37 °C at 200 rpm for a maximum of 480 min. After 60 min, 60 µL of the mixture were transferred to a reaction tube and the reactions were terminated with the addition of 20 µL ice-cold acetonitrile. The remaining mixture was incubated for a further 7 h and then stopped by adding 30 µL of ice-cold acetonitrile. After precipitation for 30 min at −18 °C, they were centrifuged for 2 min at 18,407× *g*, and 60 µL of the upper phase were transferred into an MS vial, from which 1 µL was injected into the HPLC-HRMS/MS system. To assure the absence of interfering compounds as well as to identify compounds that did not originate from the metabolism, blank incubations without substrate and control samples without pHLS9 were prepared. A positive control, ensuring the appropriate performance of the assay, was not used.

### 2.3. Rat Urine Samples for Toxicological Detectability

Rat experiments were carried out in accordance with the German Animal Welfare Act (§11 Abs. 1 TierSchG) and are here reported according to ARRIVE guidelines [13]. The protocol was adopted from previous studies using rat urine for toxicological and diagnostic purposes [14,15]. Since reference material was not available at the time of the conduction of the experiments, rats were administered the obtained drug products.

Few trip reports exist that allow the estimation of an animal equivalent dose. Troparil was reported to be consumed in doses of 20–30 mg without specification of body weight, and indatraline was reported to be consumed in doses of 25 mg, also without specification of body weight [16,17]. Assuming a body weight of 70 kg, the equivalent animal dose for troparil would be 2.0 mg/kg for the used 0.4 kg rat according to Sharma and McNeill [18]. For Indatraline, 1.9 mg/kg was calculated. Although the troparil report described nasal administration, an oral administration to rats was preferred due to ethical reasons.

The experiment consisted of the administration of indatraline or troparil to one male Wistar rat (Charles River, Sulzfeld, Germany) with 0.4 kg body weight and 4 months of age, respectively. Both drugs were administered orally with a single dose of 2 mg/kg. Each rat served as their own negative control, with six weeks between negative control sampling and drug sampling, according to the accepted study protocol. Both rats were tightly monitored after the administration, to recognize pain or suffering and to take measures, if necessary. Blinding or randomization was not used. Each rat was housed in a metabolism cage for 24 h with water ad libitum. Urine samples were collected separately from feces over a 24 h period. Inclusion criteria for each rat was the absence of recognizable pain or suffering and the excretion of at least 40 mL urine. Samples were tested directly after preparation, followed by storage at −20 °C. The urine of each rat was assessed for the detection of indatraline or troparil metabolites. Due to the qualitative character of this study, no statistical methods were used during evaluation of the samples.

### 2.4. Urine Sample Preparation

The preparation of the samples was carried out according to Wissenbach et al. [19]. An aliquot of 100 μL of the rat urine sample was mixed with 500 μL acetonitrile and vortexed for 2 min. The sample was then centrifuged at 18,407× *g* for 2 min, the supernatant was evaporated to dryness under a gentle nitrogen stream at 70 °C, and the residue was reconstituted with a 1:1 (*v/v*) mixture of 2 mM aqueous ammonium formate plus 0.1% formic acid and 2 mM aqueous ammonium formate with acetonitrile:methanol (50:50, *v/v*; 1% water) plus 0.1% formic acid. A 5-μL aliquot was injected into the Orbitrap-based HPLC-HRMS/MS as described below.

### 2.5. HPLC-HRMS/MS Apparatus

In accordance with published studies [20], this analysis was performed by using a Thermo Fisher Scientific (TF, Dreieich, Germany) Dionex UltiMate 3000 RS pump that consists of a degasser, a quaternary pump, and an UltiMate autosampler coupled to a TF Q Exactive Plus system equipped with a HESI-II heated electrospray ionization source. The final gradient elution was performed on a TF Accucore Phenyl-Hexyl column (100 mm × 2.1 mm, 2.6 µm). For the mobile phases, 2 mM aqueous ammonium formate plus 0.1% formic acid (pH 3, eluent A) and 2 mM aqueous ammonium formate with acetonitrile:methanol (50:50, *v/v*; 1% water) plus 0.1% formic acid (eluent B) were used. The flow rate was set to 0.5 mL/min for 10 min and 0.8 mL/min from 10 to 13.5 min, using the following gradient: 0–1.0 min 99% A, 1–10 min to 1% A, 10–11.5 min holding 1% A, 11.5–13.5 min holding 99% A. The following conditions applied to the HESI-II source: sheath gas, 60 arbitrary units (AU); auxiliary gas, 10 AU; spray voltage, 3.00 (positive polarity) and -4.00 kV (negative polarity); heater temperature, 320 °C; ion transfer capillary temperature, 320 °C; and S-lens RF level, 60.0. Mass spectrometry was performed by using either the positive (for indatraline and troparil) or negative (indatraline only) ionization mode via full scan (FS) data and a subsequent data-dependent acquisition (DDA) mode.

Settings for FS data acquisition were as follows: resolution, 35,000 at *m/z* 200; microscans, 1; automatic gain control (AGC) target, 1e6; maximum injection time (IT), 120 ms; and scan range, *m/z* 130–930. General screening HCD experiments were performed on the five most intense precursor ions selected from the FS using DDA and an inclusion list. The inclusion list contained *m/z* values of metabolites which were likely to be formed such as hydroxy, oxo, carboxy, and dealkyl metabolites (phase I), as well as sulfates and glucuronides (phase II) and combinations of them. The *m/z* of their [M + H]^+^ adduct was obtained after drawing them using a structure drawing software (ChemSketch, ACD/Labs, Toronto, ON, Canada). The five most intense precursor ions were included in an exclusion list for 1 s (dynamic exclusion). The remaining settings for the DDA mode were as follows: resolution, 17,500 at *m/z* 200; microscans, 1; AGC Target, 2 × 10^5^; maximum IT, 250 ms; isolation window, 1.0 *m/z*, HCD with stepped normalized collision energy, 17.5, 35, and 52.5%; spectrum data type, profile; and underfill ratio, 0.5%. Corresponding to a signal with an abundance of 4 × 10^3^, the option to exclude isotopes was enabled. TF Xcalibur Qual Browser software version 3.0.63 was used for data processing.

## 3. Results

### 3.1. Drug Product Analysis

The structural formula of methyl 3,5-dinitrobenzoate, which was used as an internal Standard is depicted in Figure S3. HPLC-HRMS/MS analysis of Indatraline and Troparil revealed, that Troparil was a mixture of two compounds with their protonated parent ions at *m/z* 246.1489 and *m/z* 260.1645 (see Figure S4). For easier interpretation of NMR results of troparil, its numbering of carbon atoms is depicted in Figure S5. Indatraline was determined in the drug product (see Figure S6, Figure S7, Figure S8, Figure S9) with qNMR as acetate salt with 38.2% purity in addition to considerable amounts of ammonium acetate. Troparil was present in the drug product as the main compound in a mixture together with its O-demethoxy derivative (see Figure S10, Figure S11, Figure S12, Figure S13, Figure S14, Figure S15, Figure S16). Their amounts were 49.3% and 27.5%, respectively. Further details are available in Supplementary Materials.

### 3.2. Tentative Identification of Metabolites

**Figure 2:**
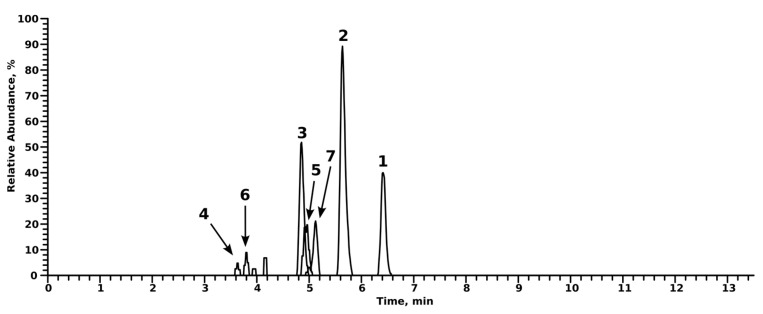
Extracted ion chromatograms of indatraline and its metabolites. Compounds are numbered according to their *m*/*z* and retention time. Normalization level is 3.5 × 105 counts.

**Figure 3:**
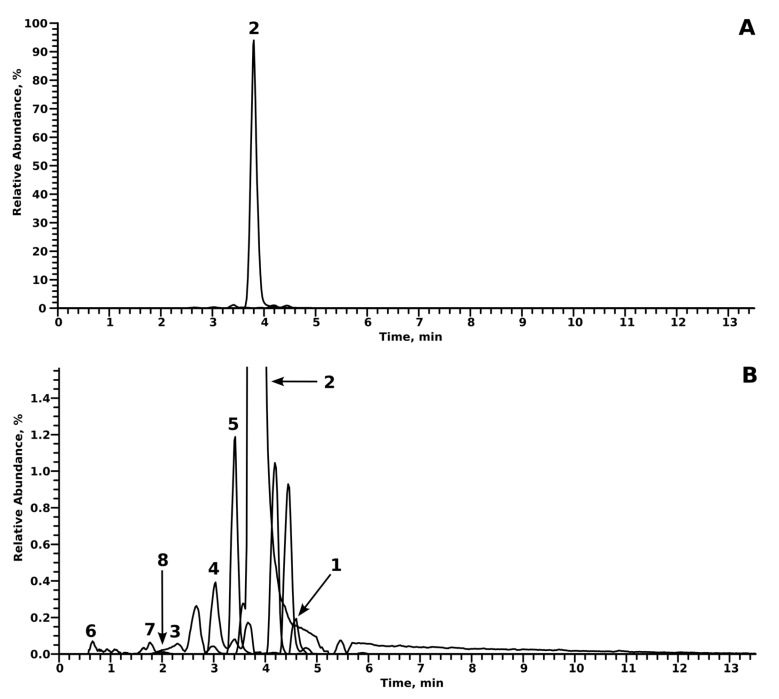
Extracted ion chromatograms of troparil and its metabolites. Compounds are numbered according to their *m*/*z* and retention time. Normalization level is 8.0 × 108 counts. (**A**) Full scale and (**B**) Zoom in to 0–1.5%.

An overview of LC-HRMS/MS data of the parent compounds and their tentatively identified metabolites is provided in Table 1 and Table 2. Metabolites were identified by comparing the spectra of the tentative metabolite with those of the parent compound. Additionally, since the abundance of indatraline and its metabolites were quite low, extracted ion chromatograms (EICs) of the parent compound and the corresponding ^37^Cl-isotope were used to reliably determine tentative peaks that might resemble indatraline or one of its metabolites. In every experiment, either blank urine or blank incubations were checked for tentative metabolites, in order to rule out spontaneous formation during the experiment. Unless otherwise stated, no analyte was found in blank urine or incubations. Chromatograms of the metabolites of indatraline and troparil can be found in Figure 2 and Figure 3, respectively.

The protonated parent ion of indatraline was detected at *m/z* 292.0654 (C_16_H_16_NCl_2_) with a retention time of 6.41 min (number 1 in Figure S1). The elimination of the secondary amine moiety led to the formation of the fragment ion at *m/z* 261.0232 (C_15_H_11_Cl_2_). Subsequently, each of the chlorine atoms was eliminated which led to the formation of the fragment ions at *m/z* 226.0544 (C_15_H_11_Cl) and *m/z* 191.0855 (C_15_H_11_). At last, the elimination of the remaining phenyl ring led to the detection of the indane ring at *m/z* 115.0621 (C_9_H_7_). The unfragmented parent ion was merely detected with a low relative abundance, which indicates that the elimination of the amine moiety was highly preferred.

The protonated parent ions of the hydroxy metabolites (number 2 and 3 in Figure S1) were detected at *m/z* 308.0604 (C_16_H_15_NOCl_2_). The increase in their parent ion masses by *m/z* 15.9949 implied the introduction of an oxygen atom. Their general fragmentation followed the same patterns as that of the parent compound. After elimination of the amine moiety and formation of the fragment ion with *m/z* 277.0187 (C_15_H_11_OCl_2_), the elimination of the chlorine atoms resulted in the formation of the fragment ions with *m/z* 242.0498 (C_15_H_11_OCl) and *m/z* 207.0805 (C_15_H_11_O), as well as the indane ring at *m/z* 115.0621 (C_9_H_7_). The observed fragmentation did not lead to a conclusive position of the hydroxy group within the structure. The occurrence of the fragment ion with *m/z* 115.0621 (C_9_H_7_) does not usually lead to a tentative placement of a hydroxy group at the aromatic part of the indane ring. However, a hydroxylation of the di-chlorinated phenyl ring appeared to be much more unlikely, due to chlorine being a deactivating substituent. Additionally, an aliphatic hydroxylation of the indane ring might have led to an initial elimination of water and a stabilization of the amine moiety by forming a hydrogen bond. Glucuronidation (number 5, 6, and 7 in Figure S1) was assumed by an increase in the observed *m/z* by 177.0393 (C_6_H_8_O_6_). After the elimination of glucuronic acid, the fragmentation continued in the same way as the corresponding phase I metabolite.

The protonated parent compound of troparil (number 1 in Figure S2) was detected at *m/z* 260.1645 (C_16_H_22_NO_2_). An initial elimination of the methoxy group led to the formation of the fragment ion with *m/z* 228.1383 (C_15_H_18_NO). The elimination of the remaining oxygen from the ester group led to the formation of the fragment ion with *m/z* 210.1277 (C_15_H_16_N). A double alkyl scission of the tropane substructure led to the formation of both the fragment ions at *m/z* 129.0699 (C_10_H_9_) and at *m/z* 84.0808 (C_5_H_10_N).

### 3.3. Proposed Metabolic Pathways of Indatraline and Troparil Drug Product in Rat and pHLS9

**Figure 4:**
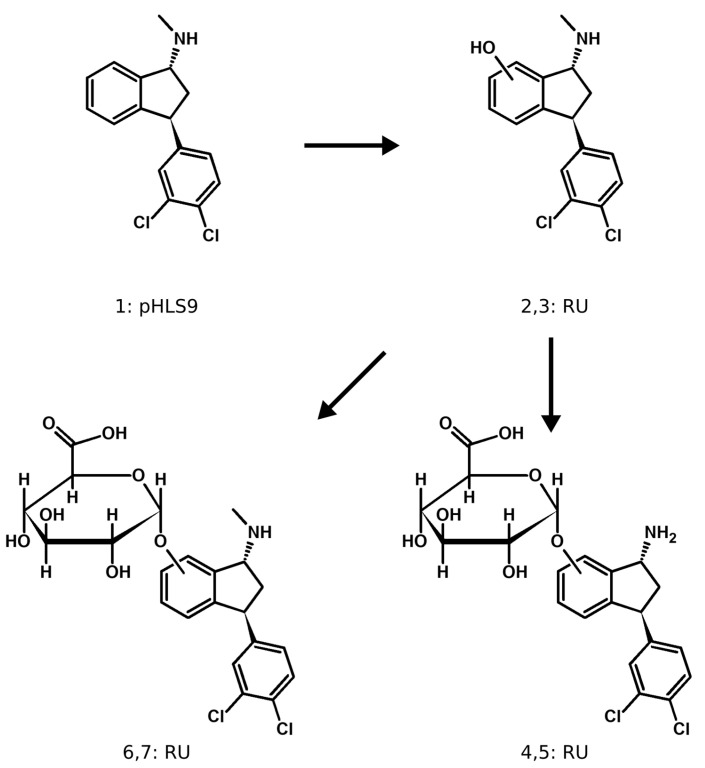
Metabolic pathways of indatraline in rat and pHLS9. Compounds are numbered according to their *m/z* and retention time. RU = detection in rat urine and pHLS9 = detection in pooled human liver S9 fraction.

The metabolic pathways of indatraline and troparil are displayed in Figure 3 and Figure 4, respectively. Indatraline itself was not detectable in rat urine but formed three phase I and four phase II metabolites, while merely the parent compound was detected in pHLS9 (number 1 in Figure 4). Hydroxylation was detected at the aromatic ring system of the indane ring (number 2 and 3 in Figure 4) which also led to the detection of corresponding glucuronides (number 6 and 7 in Figure 4). No sulfates were detected for indatraline. Although often described in the literature for secondary amines in pHLS9 and pHLM, *N*-dealkylation was not observed [11,21,22]. However, two isomers formed after demethylation, hydroxylation and glucuronidation, were detected.

**Figure 5:**
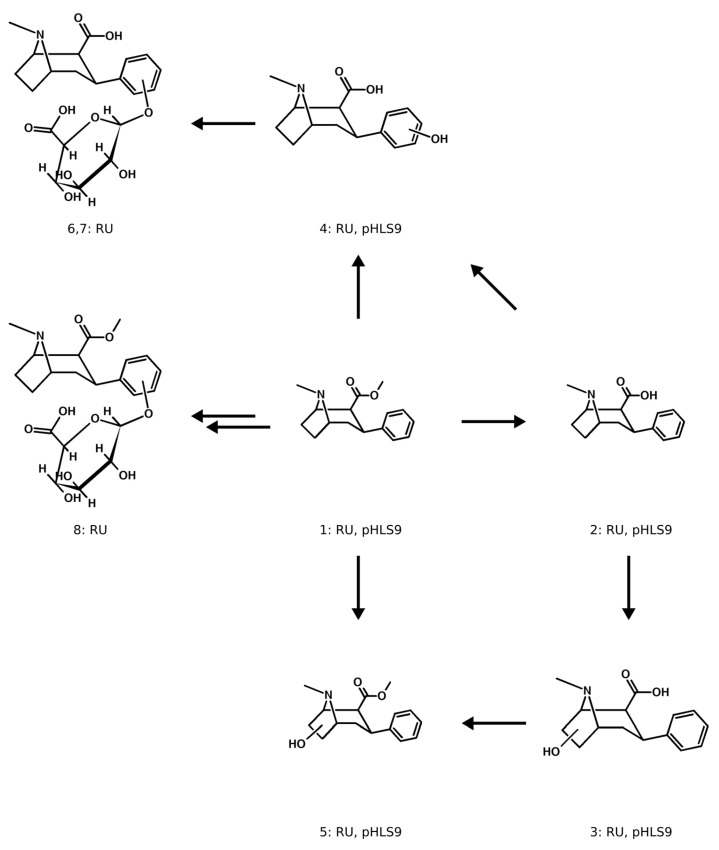
Metabolic pathways of troparil in rat and pHLS9. Compounds are numbered according to their *m/z* and retention time. RU = detection in rat urine and pHLS9 = detection in pooled human liver S9 fraction.

Troparil formed four phase I metabolites and three phase II metabolites. One of the main metabolic reactions for troparil was the hydrolysis of the ester group (number 2 in Figure 5) and hydroxylation (number 5 in Figure 5). Although the hydrolysis of the ester group metabolite was also detected in the pure substance analysis and blank incubations, the abundance in those incubations containing pHLS9 was a factor of ten higher, which is why it was considered a valid metabolite. A combination of both reactions was observed (number 4 in Figure 5), as well as hydrolyzation and aromatic hydroxylation (number 4 in Figure 5). While all phase I metabolites were detected in incubations using pHLS9 and rat urine, phase II metabolites were only detected in rat urine. Glucuronidation was observed after aromatic hydroxylation (number 8 in Figure 5), as well as after aromatic hydroxylation in combination with ester hydrolyzation (number 6 and 7 in Figure 5). A metabolite formed after aromatic hydroxylation without additional ester hydrolysis was not found. Sulfates were also not detected for troparil.

## 4. Discussion

The characterization of indatraline revealed an unexpectedly low concentration of the main compound. While other studies that investigated seized products reported powder purities between 20 and 100%, their mean determined purity was 48.6% or 81% [23,24]. Especially, the considerable amount of ammonium acetate in the indatraline drug product was, to our knowledge, not described before. This is very likely due to the fact that salts are usually not detectable using hyphenated mass spectrometry, and NMR analyses of seized powders are rarely performed. While most studies focus on pharmacologically active adulterants such as levamisole in cocaine or paracetamol in heroin, another substantial threat to the health of NPS consumers might be posed by adulterants of inorganic or other residual origin [25]. Lead however, as an inorganic adulterant, has already been described in one opium case and was linked to non-specific symptoms such as nausea and abdominal pain [25].

The HPLC-HRMS/MS method used in this study is not meant to provide a ready-to-use quantification method for the investigated compounds. Therefore, no validation concerning selectivity, linearity, or response was conducted. However, the fit of the applied HPLC-HRMS/MS method to this study was already shown by Richter et al., as well as numerous other publications that also applied it to incubations using pHLS9 and rat urine in order to elucidate the metabolism of NPSs [11,12,26,27,28,29].

Although low in concentration, the amount of main compound in the troparil drug product was within the expected range. Regarding the NMR-analysis of troparil, the occurrence of *O*-demethyl-troparil is quite likely the result of an insufficient synthesis yield. The remaining 23.2% of the powder was not determinable with LC-HRMS/MS or NMR.

The reason for absence of any indatraline metabolites in pHLS9 might be the lower enzyme activity of cytochrome P450 enzymes in pHLS9 compared to other metabolism models, such as pooled human liver microsomes (pHLM) [30]. Additionally, a concentration of metabolites in urine, likely occurring in living beings such as rats, is not possible [31]. A reason for the missing metabolite formed after *N*-dealkylation might be a rather quick catalyzation of the glucuronidation reaction, leaving the corresponding phase I metabolites in concentrations below the limit of detection. Further enzyme kinetic studies are necessary to investigate this theory. Due to the high structural resemblance of indatraline to sertraline, it is of interest to compare their metabolism. Sertraline is reported to excrete an alpha-ketone glucuronide metabolite into the urine of male Prague-Dawley rats, as well as a carbamoyl glucuronide, an *N*-hydroxy glucuronide, and an alpha-ketone glucuronide into the urine of beagle dogs [32]. Additionally, the *N*-demethyl metabolite and a ketone are described to be the main metabolites in humans [33,34]. None of these metabolites were found in pHLS9 or rats, which might be due to interspecies differences of CYP enzymes concerning expression, activity, or substrate affinity [35,36].

However, the metabolism studies of troparil lead to the detection of several metabolites in rat urine as well as in pHLS9. Since several metabolites were formed after ester hydrolysis of troparil, it is likely that this reaction is quickly catalyzed, and thus the remaining product was likely below the limit of detection. The reason for not detecting phase II metabolites in incubations using pHLS9 might be the generally lower substrate affinity of UDP-glucuronosyl transferases [37]. Comparing the metabolism of troparil to the metabolites found for its archetype cocaine in human plasma and urine, both show limited parallels [38]. Like cocaine, the main metabolic step of troparil is demethylation, while a combination of demethylation and aromatic hydroxylation is well described in the literature [38]. However, other important metabolic steps of cocaine are *N*-demethylation and hydrolysis of the benzoate ester. While the first one was not observed for troparil, it does not contain a benzoate ester that might have been hydrolyzed. On the other side, tropane ring hydroxylation and glucuronidation has not been reported for cocaine. It is notable that the detection of a transesterification product of troparil resulting in an ethyl ester is likely when alcohol is co-consumed [38].

## 5. Conclusions

This study investigated the purity of an indatraline and troparil drug product, as well as the metabolism of the two compounds after administration to rats and incubations using pHLS9. While the purity of indatraline was determined to be 38.2%, the purity of troparil was 49.3%, with both drug products containing significant adulterations. Indatraline formed two metabolites after hydroxylation at the indane ring and four glucuronides, two after hydroxylation of the indane ring and two after combination of hydroxylation and demethylation. All metabolites were detected in rat urine and none in incubations using pHLS9. The main metabolic step for troparil was demethylation of the ester group. This led to the formation of one demethylated metabolite, two metabolites after demethylation and hydroxylation, as well as one after demethylation, hydroxylation, and glucuronidation. Additionally, single hydroxylation and hydroxylation in combination with glucuronidation was detected. Comparing the metabolic pathway of indatraline to sertraline merely showed a common demethylation, while troparil and cocaine share ester hydrolyzation and aromatic hydroxylation. Due to the lack of metabolites detected in pHLS9, especially for indatraline, further data from pHLM or hepatocytes should be collected, to complement the results provided in this study and provide further insights into human metabolism. This study provided the first tentative targets for clinical and forensic toxicological analyses. The absence of the indatraline parent compound in rat urine might hint to the importance of including metabolites as screening targets. Concerning troparil, however, screening procedures may focus on the detection of demethylated metabolites, since they were the most abundant signals, and it is likely that they will be the most abundant in human samples as well.

## Figures and Tables

**Table 1:** Overview of indatraline and its metabolites with their detected *m*/*z* of the protonated parent ion from MS1, retention time, chemical formula, and their mass error in ppm. Metabolites are sorted by *m*/*z* and retention time.

Analyte	[M + H], *m*/*z*	Retention Time, min	Chemical Formula	ΔMass, ppm
Indatraline	292.0647	6.41	C_16_H_16_NCl_2_	−2.4
Indatraline-M (HO-) Isomer 1	308.0600	5.04	C_16_H_16_NOCl_2_	−1.0
Indatraline-M (HO-) Isomer 2	308.0607	5.64	C_16_H_16_NOCl_2_	1.3
Indatraline-M (Demethyl-HO-) Glucuronide Isomer 1	470.0776	3.54	C_21_H_22_NO_7_Cl_2_	−1.7
Indatraline-M (Demethyl-HO-) Glucuronide Isomer 2	470.0758	4.79	C_21_H_22_NO_7_Cl_2_	−2.0
Indatraline-M (HO-) Glucuronide Isomer 1	484.0927	4.02	C_22_H_24_NO_7_Cl_2_	−0.6
Indatraline-M (HO-) Glucuronide Isomer 2	484.0918	4.87	C_22_H_24_NO_7_Cl_2_	−1.4

**Table 2:** Overview of troparil and its metabolites with their detected *m*/*z* of the protonated parent ion from MS1, retention time, chemical formula, and their mass error in ppm. Metabolites are sorted by *m*/*z* and retention time.

Analyte	[M + H], *m*/*z*	Retention Time, min	Chemical Formula	ΔMass, ppm
Troparil	260.1638	4.25	C_16_H_22_NO_2_	−2.6
Troparil-M (Demethyl-)	246.1481	3.78	C_15_H_20_NO_2_	−3.0
Troparil-M (Demethyl-HO-) Isomer 1	262.1433	2.30	C_15_H_20_NO_3_	−1.6
Troparil-M (Demethyl-HO-) Isomer 2	262.1429	3.02	C_15_H_20_NO_3_	−3.3
Troparil-M (HO-)	276.1585	3.40	C_16_H_22_NO_3_	−3.3
Troparil-M (Demethyl-HO-) Glucuronide Isomer 1	438.1747	0.63	C_21_H_28_NO_9_	−2.8
Troparil-M (Demethyl-HO-) Glucuronide Isomer 1	438.1751	1.76	C_21_H_28_NO_9_	−1.8
Troparil-M (HO-) Glucuronide	452.1919	2.01	C_22_H_30_NO_9_	0.9

## Data Availability

The datasets presented in this article are not readily available because they are part of an ongoing study. Requests to access the datasets should be directed to the corresponding author.

## Supplementary Materials

The following supporting information can be downloaded at: https://www.mdpi.com/article/10.3390/metabo14060342/s1, Figure S1: LC-HRMS/MS spectra of indatraline and its metabolites sorted by mass of the protonated molecule and retention time, proposed chemical structure, accurate mass, calculated elemental formula, and mass error value in parts per million (ppm); Figure S2: LC-HRMS/MS spectra of troparil and its metabolites sorted by mass of the protonated molecule and retention time, proposed chemical structure, accurate mass, calculated elemental formula, and mass error value in parts per million (ppm), NMR spectroscopy: detailed description of the NMR method and instrumentation; Figure S3: Methyl 3,5-dinitrobenzoate as internal standard, Structure Characterization: detailed description of the qualitative NMR results [9,39]; Table S1: NMR data of indatraline acetate (DMSO-d_6_, ^1^H: 500 MHz, ^13^C: 125 MHz) with its structure and numbering of atoms; Figure S4: Total ion chromatogram of the troparil substance after LC-HRMS/MS and corresponding MS^2^ spectra of the major and minor compounds detected including their proposed chemical structure, accurate mass, calculated elemental formula, and mass error value in parts per million (ppm); Table S2: NMR data of troparil and *O*-demethyltroparil (DMSO-d_6_, ^1^H: 500 MHz, ^13^C: 125 MHz), Quantitative analysis (qNMR): detailed description of the quantitative NMR results [40]; Figure S6: ^1^H NMR spectrum of indatraline sample. Signals marked with an asterisk originate from the internal qNMR standard methyl 3,5-dinitrobenzoate; Figure S7: ^1^H NMR spectrum of indatraline sample, aromatic region. Signals marked with a hashtag originate from NH4+; Figure S8. 13C NMR spectrum of indatraline sample; Figure S9: ^13^C NMR spectrum of indatraline sample, aromatic region. Signals with asterisk originate from the internal qNMR standard methyl 3,5-dinitrobenzoate; Figure S10: ^1^H NMR spectrum of troparil sample. Signals marked with an asterisk originate from the internal qNMR standard methyl 3,5-dinitrobenzoate; Figure S11: ^1^H NMR spectrum of troparil sample, aliphatic region. Signal marked with an asterisk originate from the internal qNMR standard methyl 3,5-dinitrobenzoate; Figure S12. 13C NMR spectrum of troparil sample; Figure S13: ^13^C NMR spectrum of troparil sample, aromatic region. Signals marked with an asterisk originate from the internal qNMR standard methyl 3,5-dinitrobenzoate; Figure S14: ^13^C NMR spectrum of troparil sample, aliphatic region. Signal marked with an asterisk originate from the internal qNMR standard methyl 3,5-dinitrobenzoate; Figure S15: ^1^H qNMR spectrum of indatraline sample, aromatic region, with integrals for the calculation; Figure S16: ^1^H qNMR spectrum of troparil sample with integrals for the calculations.
