# Supplementary material for: Characterization and Metabolism of Drug Products Containing the Cocaine-Like New Psychoactive Substances Indatraline and Troparil
Source: Metabolites. 2024 Jun 18;14(6):342. doi: 10.3390/metabo14060342 (PMC11205633; doi:10.3390/metabo14060342)
Supplement: Supplementary file 1 [file metabolites-14-00342-s001.zip › metabolites-2994902-supplementary materials.pdf]

Investigations on the in vivo and in vitro metabolic fate of the cocaine-like new psychoactive substances indatraline and troparil

Sascha K. Manier, Paula Mumber, Josef Zapp, Niels Eckstein, Markus R. Meyer

Electronic Supplementary Material

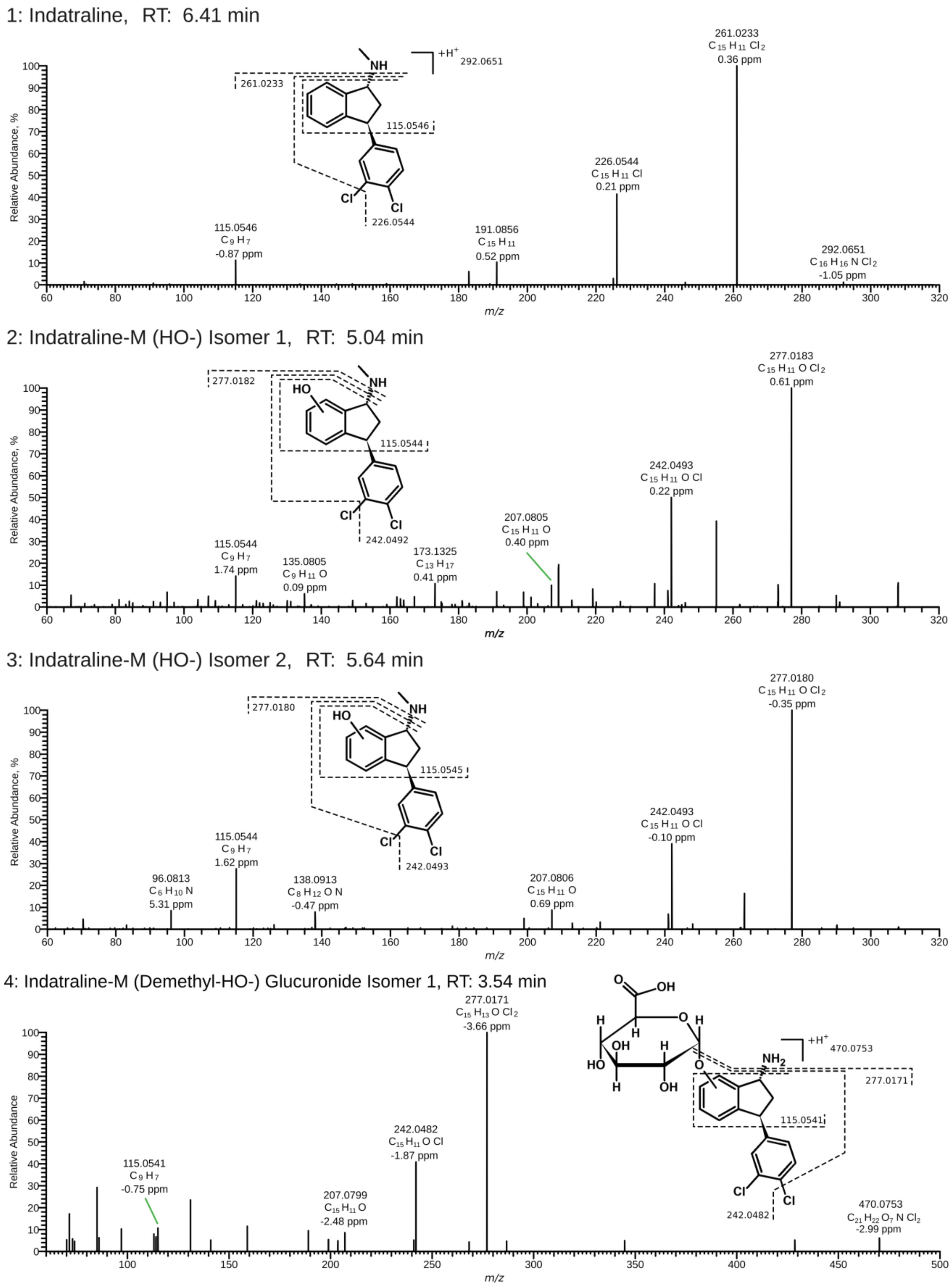

Figure S1. LC-HRMS/MS spectra of indatraline and its metabolites sorted by mass of the protonated molecule and retention time, proposed chemical structure, accurate mass, calculated elemental formula, and mass error value in parts per million (ppm).

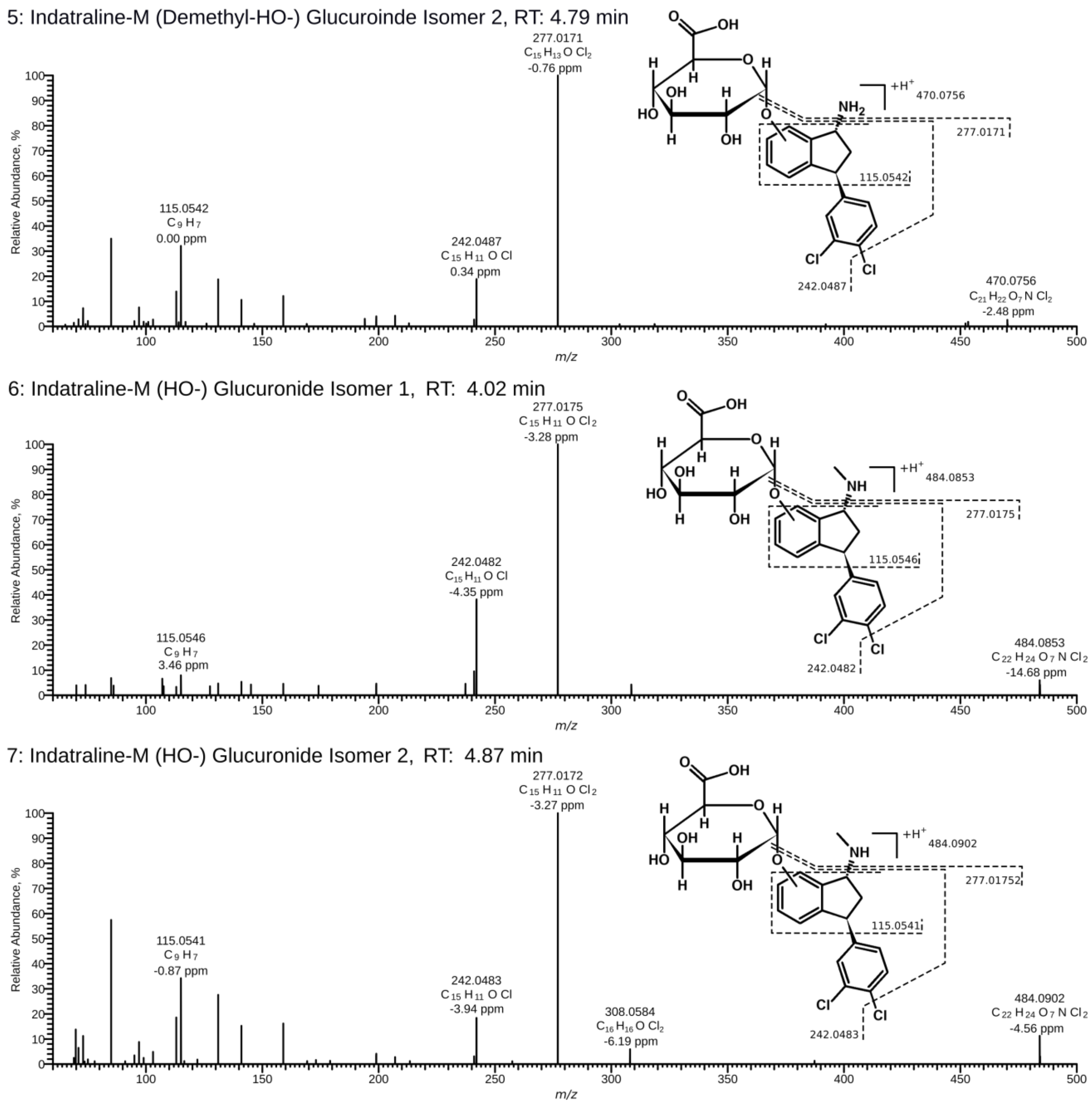

Figure S1. continued.

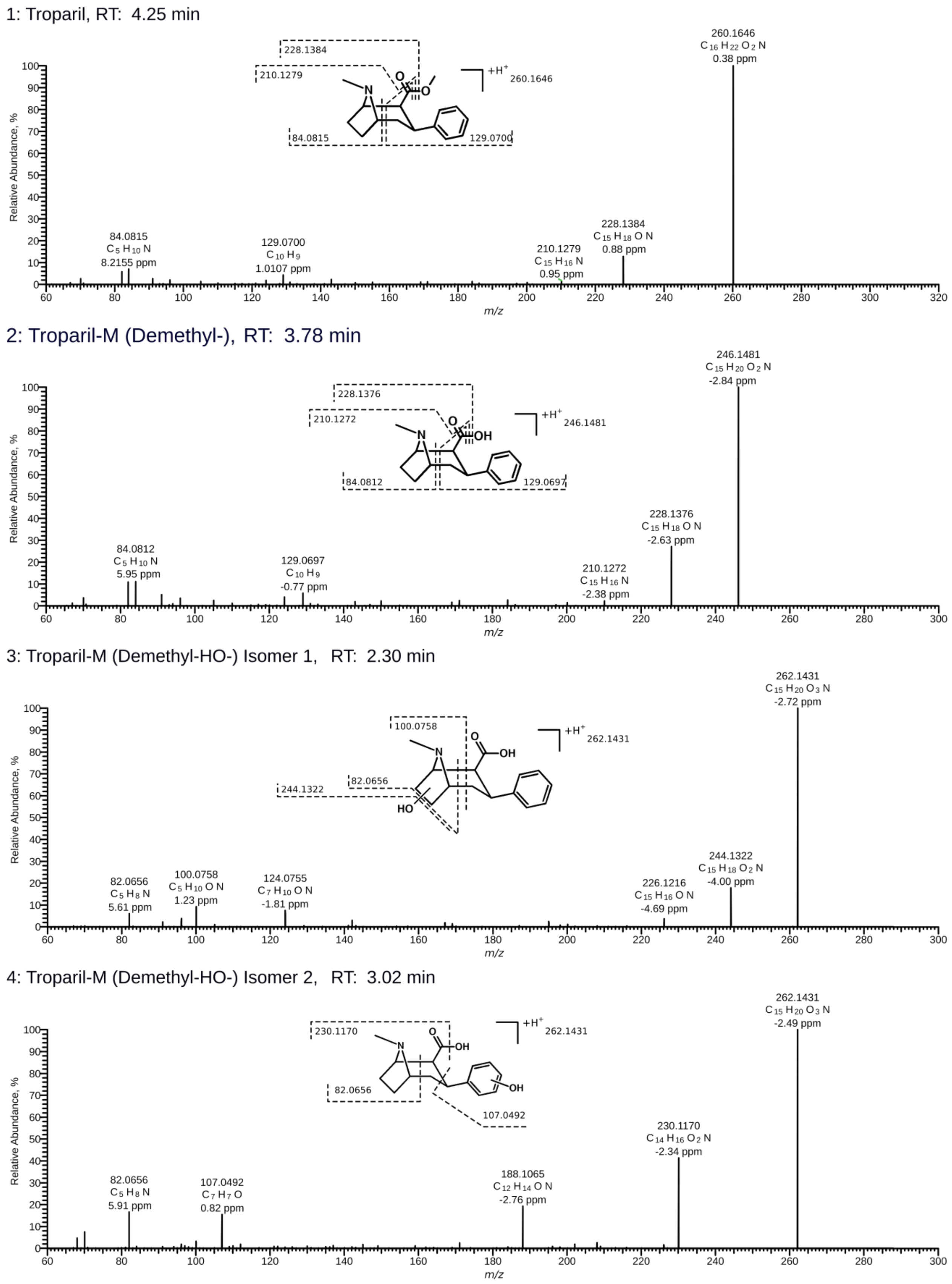

Figure S2. LC-HRMS/MS spectra of troparil and its metabolites sorted by mass of the protonated molecule and retention time, proposed chemical structure, accurate mass, calculated elemental formula, and mass error value in parts per million (ppm).

5: Troparil-M (HO-), RT: 3.40 min

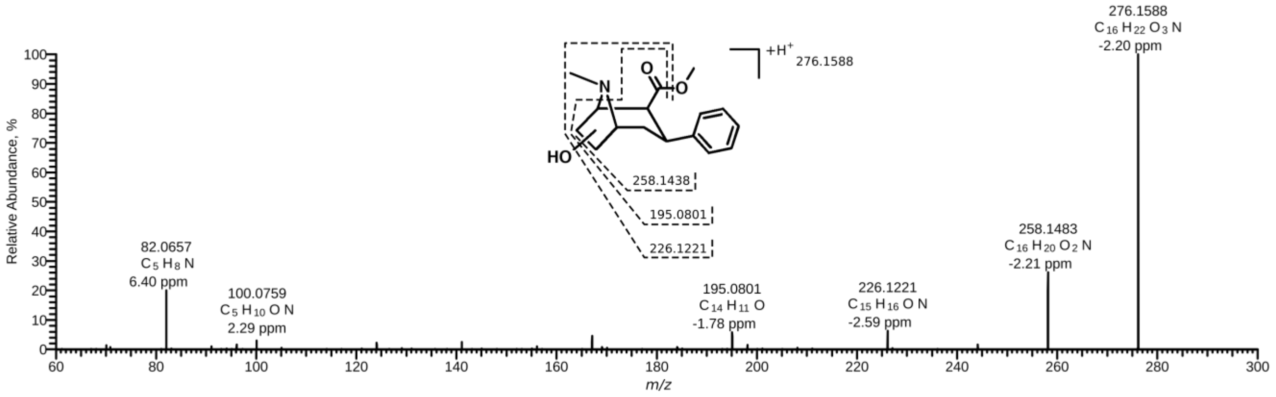

6: Troparil-M (Demethyl-HO-) Glucuronide Isomer 1, RT: 0.63 min

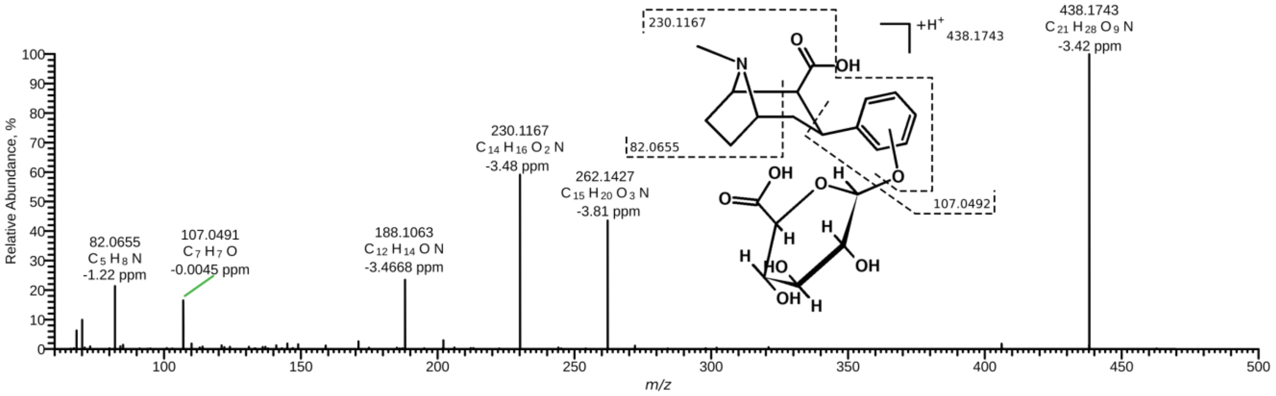

7: Troparil-M (Demethyl-HO) Glucuronide Isomer 2, RT: 1.76 min

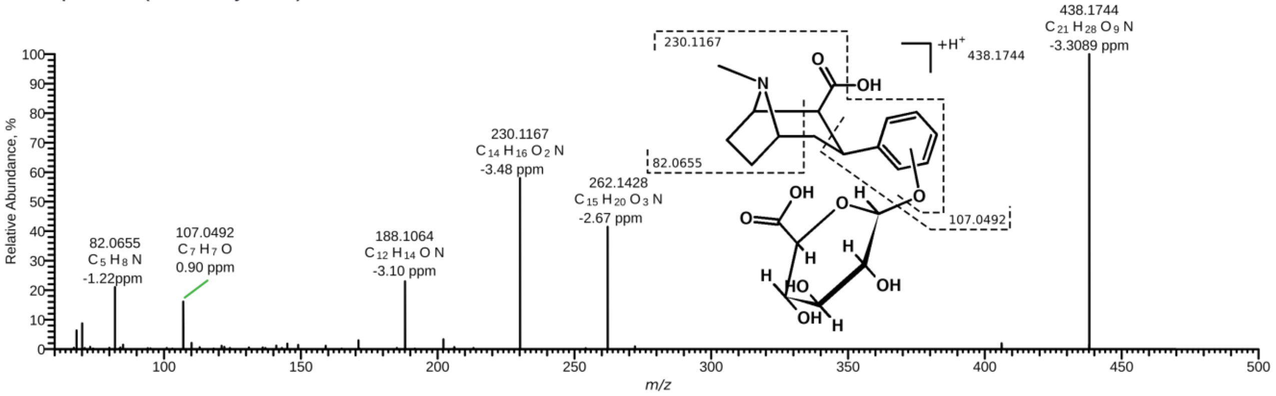

8: Troparil-M (HO-) Glucuronide, RT: 2.01 min

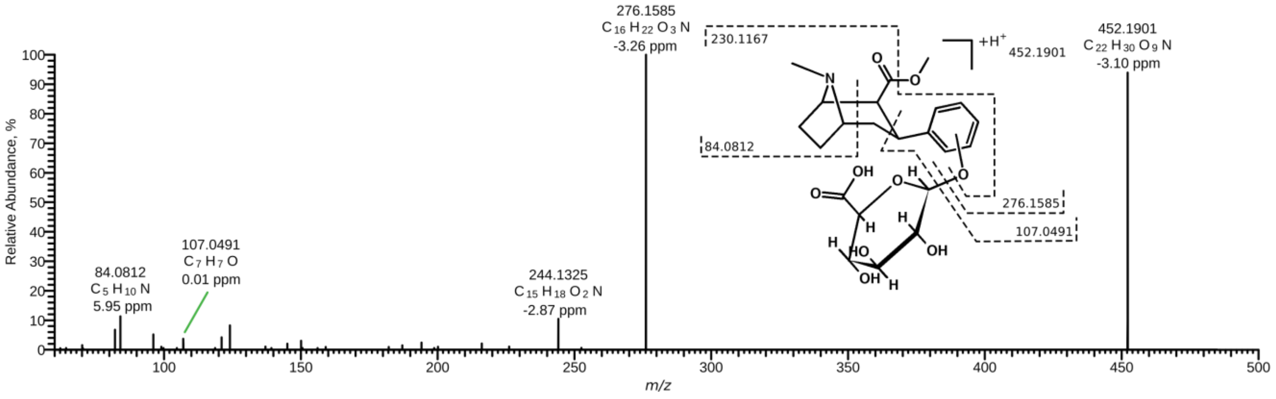

Figure S2. continued.

NMR spectroscopy

NMR spectra were recorded in DMSO-d_6 (Deutero, Kastellaun, Germany) on a Bruker Avance Neo 500 Spectrometer (Bruker Biospin, Germany) equipped with a TCI Prodigy cryoprobe at 298K. ^{1}H, ^{13}C and ^{1}H quantitative NMR spectra (qNMR) were recorded using standard pulse programs from the Bruker pulse library. NMR chemical shifts were reported in parts per million, relative to the residual solvent signal DMSO at δ 2.50 for ^{1}H measurements and δ 39.5 (DMSO-d_6) for ^{13}C measurements. NMR data were analyzed using Topspin, version 4.30 (Bruker, Biospin GmbH, Rheinstetten, Germany).

For qNMR purposes, methyl 3,5-dinitrobenzoate (purchased from Fluka, TraceCert, with a certified value (mass fraction) of 99.84 %) was added to the samples as an internal standard. Since all measurements of both samples were recorded with the qNMR sample, all spectra contain the signals of the internal standard. For a better understanding, these signals were therefore labelled with an asterisk in the spectra figures.

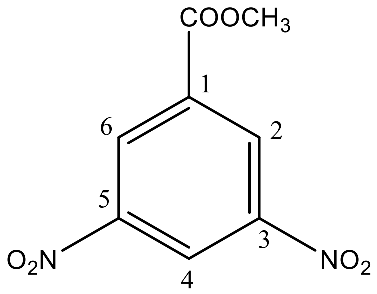

Figure S3. Methyl 3,5-dinitrobenzoate as internal standard.

Structure Characterization

Indatraline

Its molecular formula was calculated as $C_{16}H_{16}NCl_2$ based on HRMS with *m/z* 292.0654 $[M+H]^+$. ^1H and ^{13}C NMR spectra in DMSO-d_6 showed signals for a protonated N-methyl 2,3-dihydro-1*H*-indane, a 1,2,4 trisubtituted benzene and an acetate moiety. These data led to the structure of indatraline acetate (table S1), which are in good accordance with those from literature[1]. A detailed examination of the NMR spectra also revealed resonances for acetic acid (δ_C 172.01 and 21.09; δ_H 1.91, s, 3H) and ammonia (7.36, t, $J_{1H,14N}$ = 50.8 Hz) as components of ammonium acetate, which was present in considerable amounts.

	δ_C	δ_H multiplicity (J in Hz)
1	61.33	4.88 m
2	37.37	2.75 ddd (14.5, 8.2, 3.3 Hz)<br>2.43 dt (14.5, 7.7 Hz)
3	47.48	4.81 t (7.7 Hz)
3a	147.11	-------------------
4	125.15	7.03 m
5	127.52	7.39 m
6	130.08	7.38 m
7	126.45	7.82 m
7a	137.55	-------------------
1′	145.20	-------------------
2′	129.78	7.43 d (2.1 Hz)
3′	129.31	-------------------
4′	131.23	-------------------
5′	130.86	7.59 d (8.2 Hz)
6′	128.27	7.16 dd (8.2, 2.1 Hz)
NCH_3	29.92	2.56 t (5.4 Hz; 3H)
NH_2	----------	9.71 brs<br>9.63 brs
OAc	171.62<br>22.46	-------------------<br>1.77 s (3H)

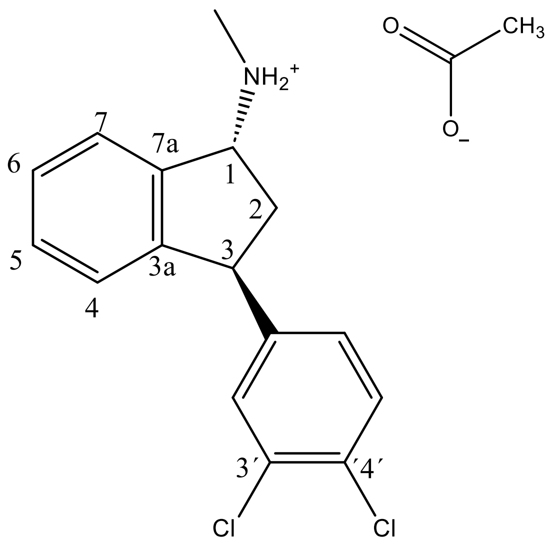

Table S1. NMR data of indatraline acetate (DMSO-d_6, ^1H: 500 MHz, ^{13}C: 125 MHz) with its structure and numbering of atoms.

Troparil

Its total ion chromatogram (TIC) showed two peaks with retention times of 3.89 min (minor component) and 4.25 min (major component) with *m/z* 246.1489 and *m/z* 260.1645, indicating a mixture of two compounds differing by one CH_2 moiety. This was consistent with the results of NMR spectroscopy, which revealed resonances of two very close related phenyltropane-derived molecules in the ^{1}H and ^{13}C NMR spectra (table S2). The data of the main compound matched perfectly with literature data of the troparil[2], those of the minor component lacked only signals for the methyl ester group and thus belonged to the free acid *O*-demethyltroparil.

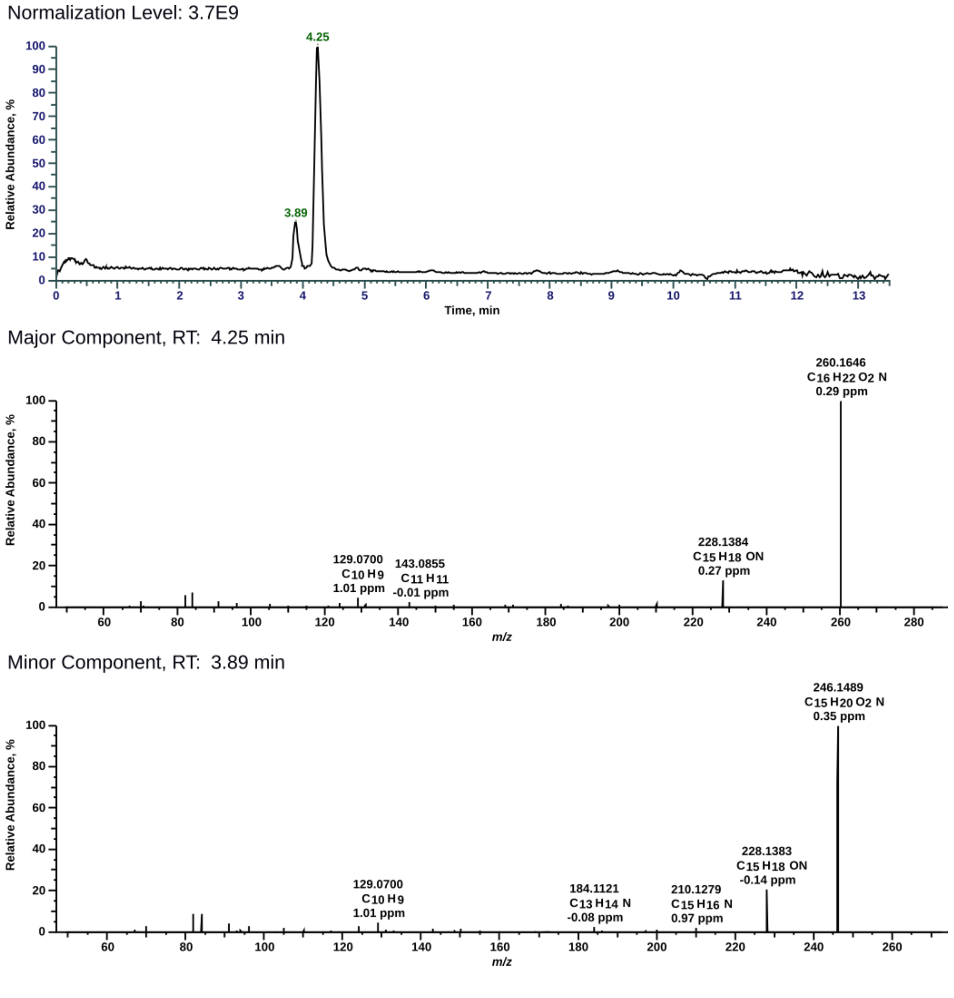

Figure S4. Total ion chromatogramm of the troparil substance after LC-HRMS/MS and corresponding MS^2 spectra of the major and minor compounds detected includind their proposed chemical structure, accurate mass, calculated elemental formula, and mass error value in parts per million (ppm).

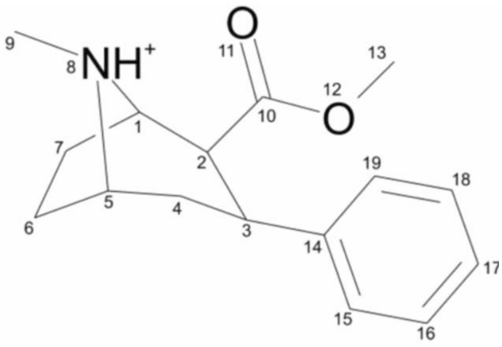

Figure S5. troparil with numbering of atoms

	Troparil		O-Demethyltroparil	
	δC	δ_H multiplicity (J in Hz)	δC	δ_H multiplicity (J in Hz)
1	62.70	4.12 m (7.5 Hz)	62.86	4.11 m (7.5 Hz)
2	51.51	3.28 dd (8.8, 1.5 Hz)	51.22	3.22 dd (8.0, 1.5 Hz)
3	33.91	3.36 m	33.87	3.36 m
4	35.10	2.47 m<br>1.94 dd (14.4, 9.2 Hz)	34.75	2.47 m<br>1.94 m
5	60.89	3.90 t (7.2 Hz)	61.08	3.86 t (7.2 Hz)
6	25.56	2.24<br>1.87	25.22	2.21<br>1.84
7	25.61	2.34<br>2.03	25.22	2.32<br>1.96
8	---------	9.44	---------	9.36
9	38.70	2.71 s (3H)	38.67	2.71 s (3H)
10	173.28	-------------------	174.07	-------------------
13	52.20	3.99 s (3H)	--------	-------------------
14	141.51	-------------------	141.94	-------------------
15/19	127.34	7.39 m	127.27	7.39 m (2H)
16/18	128.49	7.34 m	128.43	7.34 m (2H)
17	126.84	7.25 m	126.67	7.25 m
NH	---------	9.45 brs	---------	9.45 brs

Table S2. NMR data of troparil and *O*-demethyltroparil (DMSO-d_6, ^{1}H: 500 MHz, ^{13}C: 125 MHz)

Quantitative analysis (qNMR)

The purity of the analytes was analyzed via qNMR with methyl 3,5-dinitrobenzoate as internal standard . Therefore, a defined amount of the analyte was weighed (~10 mg) and dissolved in 1 ml of standard stock solution in DMSO-d_6 (101.25 mg /25 ml DMSO-d_6) and measured via qNMR in an analogous manner as described by Malz[3].

The purity of the analyte P_x was then calculated as follows:

$$P_x = \frac{I_x}{I_{\text{Std}}} \frac{N_{\text{Std}}}{N_x} \frac{M_x}{M_{\text{Std}}} \frac{m_{\text{Std}}}{m} P_{\text{Std}}$$

where M_x and M_{Std} are the molar masses of the analyte and the standard, respectively, m_X the weighed mass of the investigated sample, m_{Std} and P_{Std} are the weighed mass and the purity of the standard and N_{Std} and I_{Std} correspond to the number of spins and the integrated signal area of a (typical) NMR line of the standard. This procedure led to the following results:

Indatraline sample

As already mentioned, the sample contained significant amounts of ammonium acetate. After analysing of its qNMR spectrum indatraline was calculated as acetate to $\boldsymbol{P_{Ind}}$ **= 38.2 %**

I_{Ind} = 65.21 (H-5′ at δ_H 7.59)

N_{Ind} = 1

M_{Ind} = 351.08

m_{Ind} = 10.72

P_{Std} = 99.84

I_{Std} = 100 (H-4 at δ_H 9.04)

N_{Std} = 1

M_{Std} = 226.14

m_{Std} = 4.05

Troparil sample

Due to their very similar chemical shifts, both tropanes were initially determined as a whole. For this purpose, the integral of aromatic H-17, which appeared isochrone for both compounds, was used for calculation. Then, the *O*-methyl group, which was only present in troparil but not in its *O*-demethyl derivative, was analyzed. In this way, the amount of troparil alone and after subtraction from the total tropane amount, also that of *O*-demethyltroparil could be determined. All compounds were calculated as HCl salt.

Determination of the total tropane amount: P_{total} = 76.8 %

I_{total} = 151.2 (H-17, δ_H 7.26)	I_{Std} = 100 (H-4′ at δ_H 9.05)
N_{total} = 1	N_{Std} = 1
M_{total} = 295.81	M_{Std} = 226.14
m_{total} = 10.42	m_{Std} = 4.05
	P_{Std} = 99.84

Determination of troparil: P_{Trop} = 49.3 %

I_{Trop} = 291.0 (H-1§, δ_H 3.57)	I_{Std} = 100 (H-4 at δ_H 9.05)
N_{Trop} = 3	N_{Std} = 1
M_{Trop} = 295.81	M_{Std} = 226.14
m_{Trop} = 10.42	m_{Std} = 4.05
	P_{Std} = 99.84

Determination of *O*-demethyl troparil: P_{total} - P_{Trop} = $P_{deMeTrop}$ = *27.5%*

NMR spectra

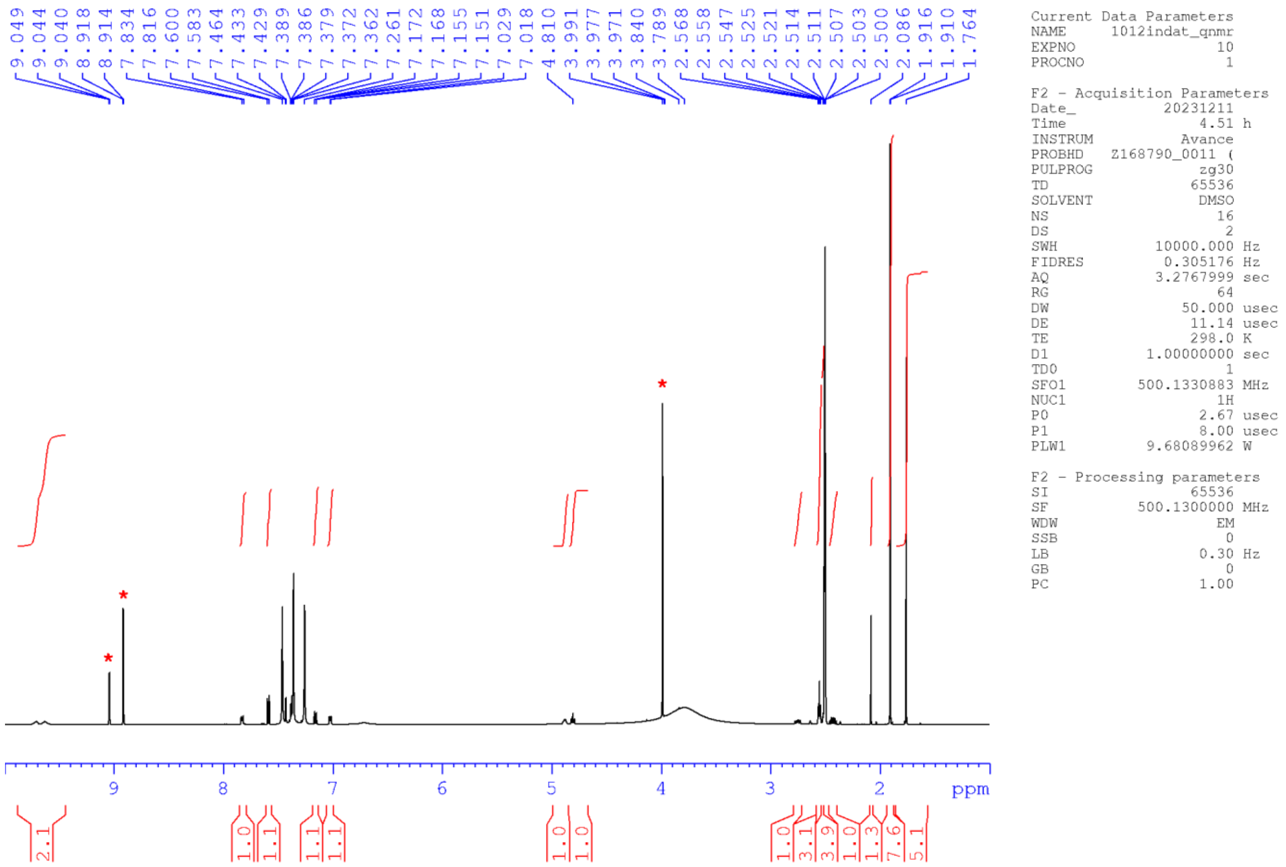

Figure S6. ^{1}H NMR spectrum of indatraline sample. Signals marked with an asterisk originate from the internal qNMR standard methyl 3,5-dinitrobenzoate.

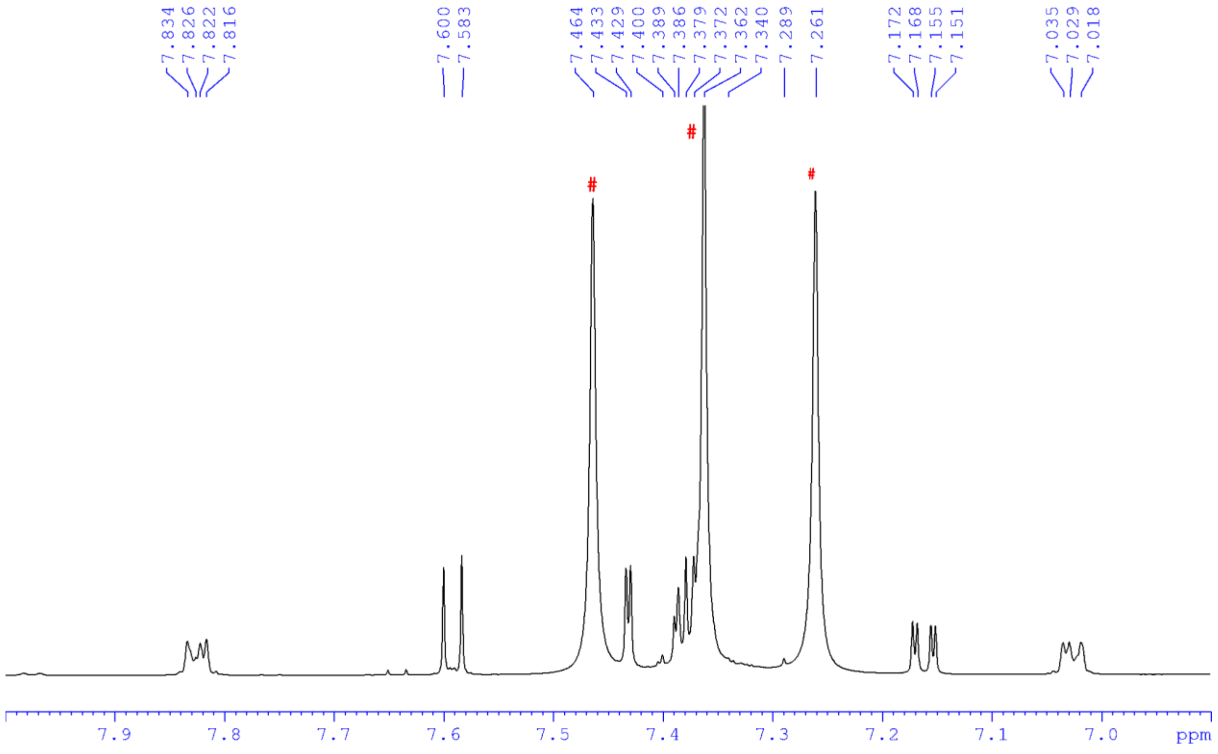

Figure S7. ^{1}H NMR spectrum of indatraline sample, aromatic region. Signals marked with a hashtag originate from NH_4^+.

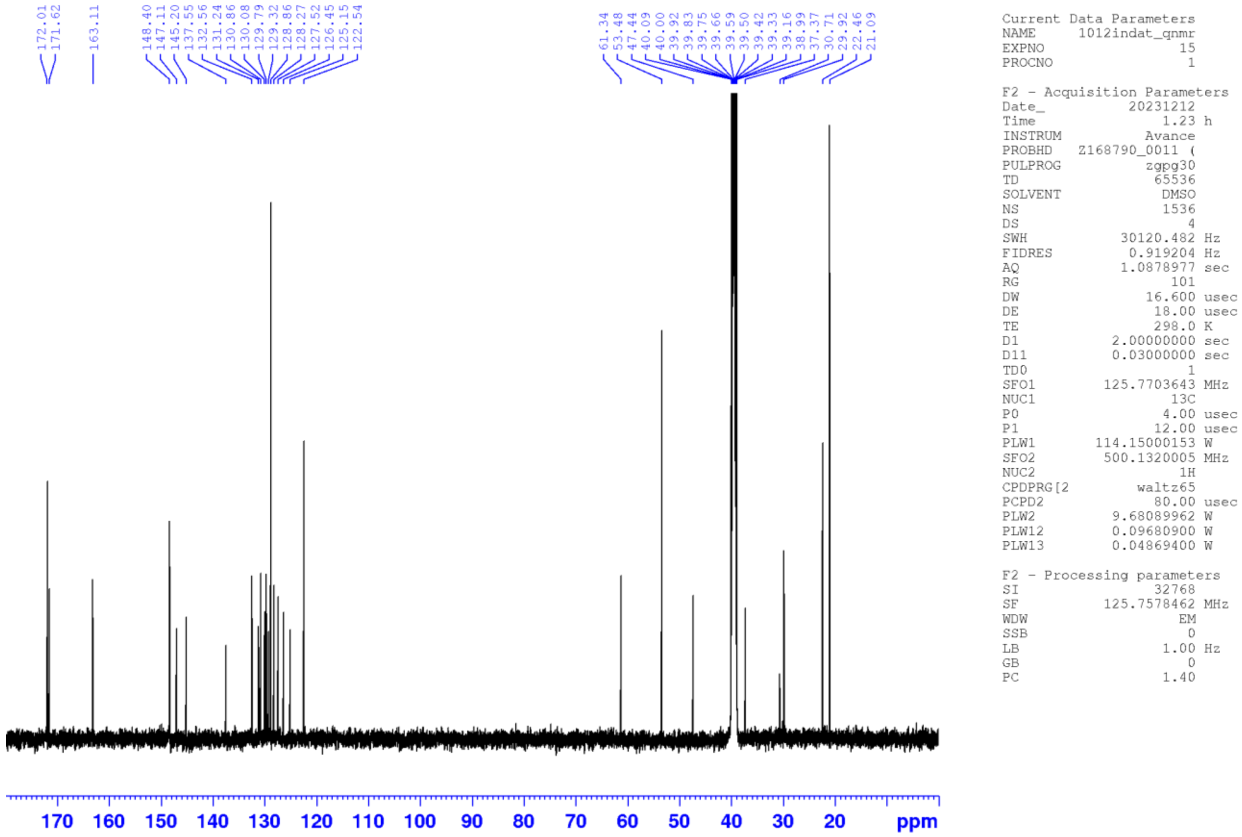

Figure S8. ^{13}C NMR spectrum of indatraline sample.

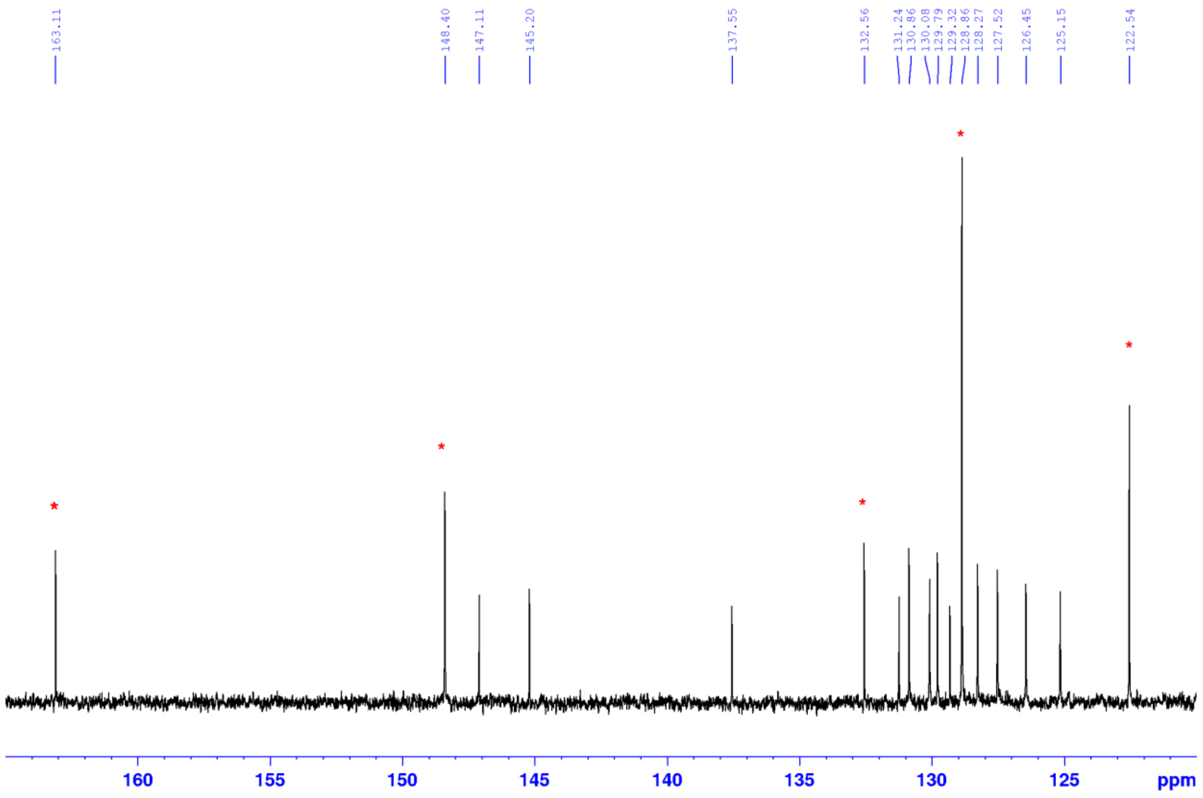

Figure S9. ^{13}C NMR spectrum of indatraline sample, aromatic region. Signals with asterisk originate from the internal qNMR standard methyl 3,5-dinitrobenzoate.

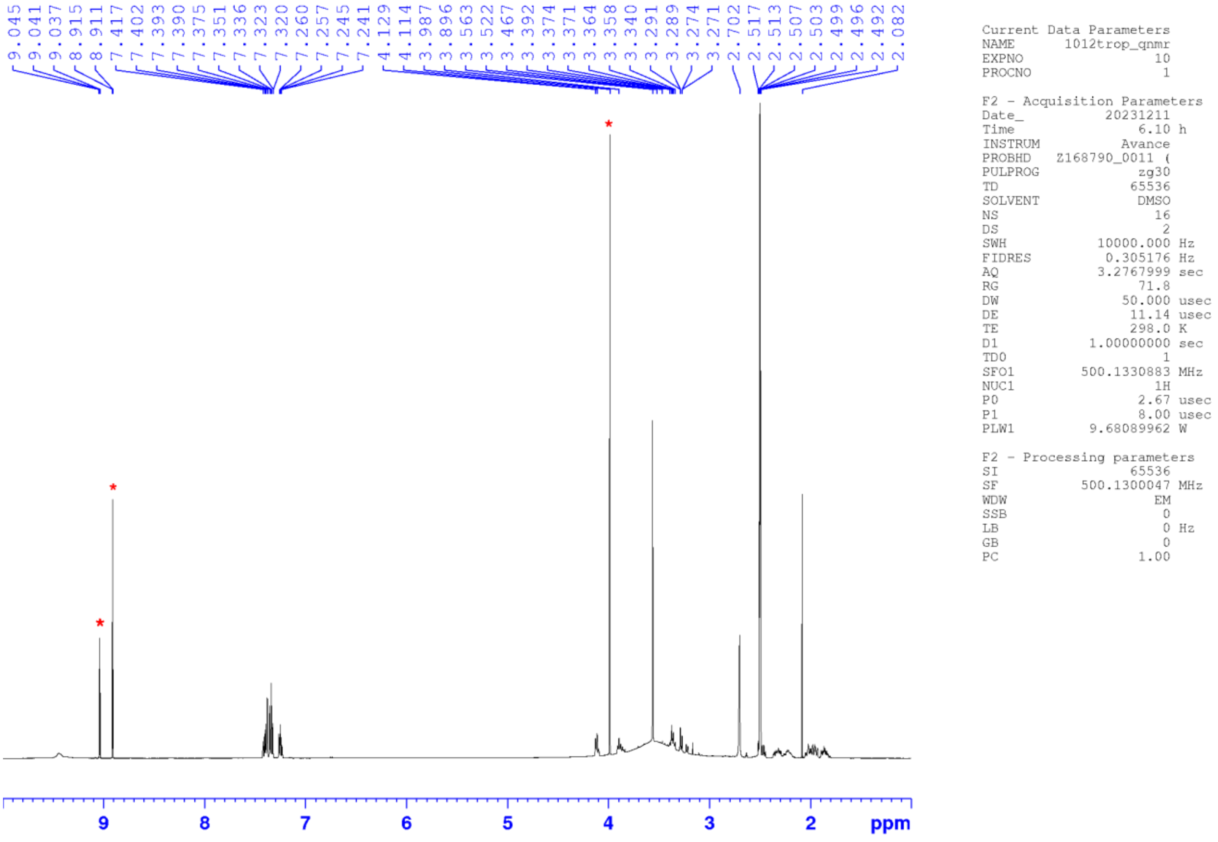

Figure S10. ^{1}H NMR spectrum of troparil sample. Signals marked with an asterisk originate from the internal qNMR standard methyl 3,5-dinitrobenzoate.

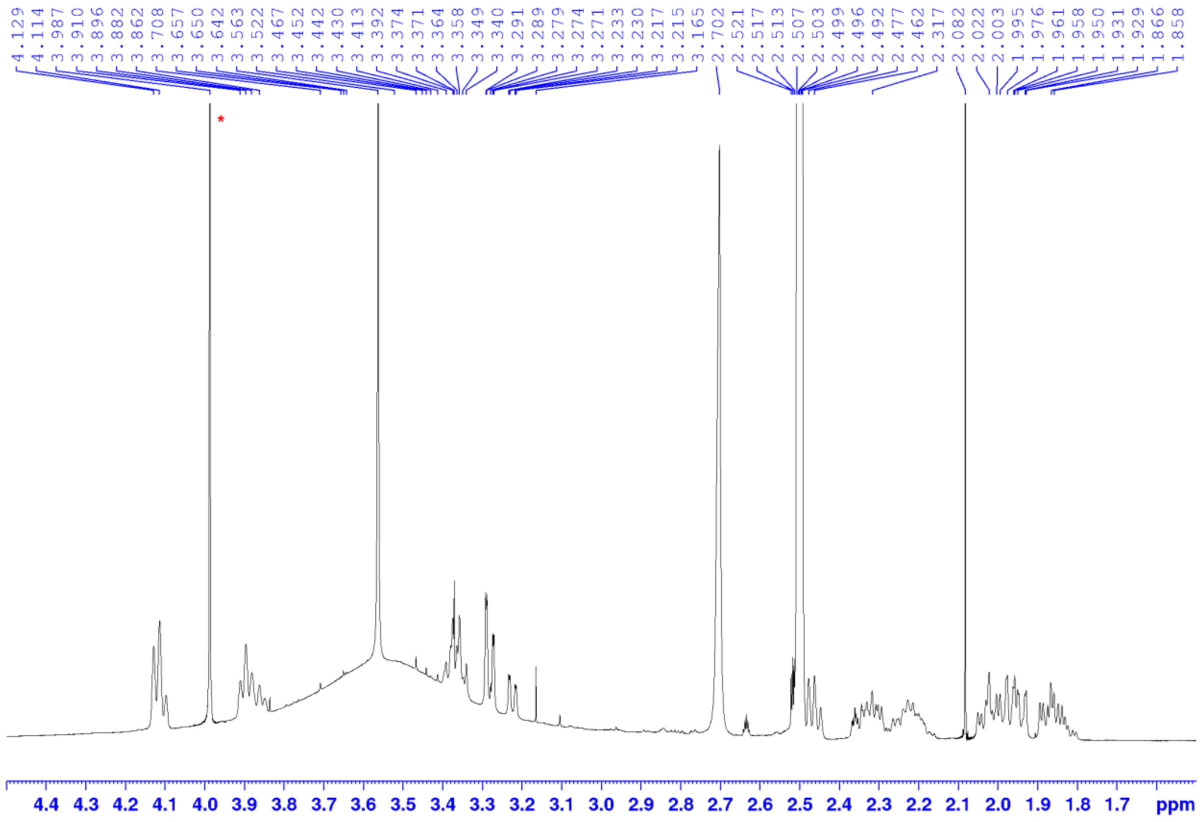

Figure S11. ^{1}H NMR spectrum of troparil sample, aliphatic region. Signal marked with an asterisk originate from the internal qNMR standard methyl 3,5-dinitrobenzoate.

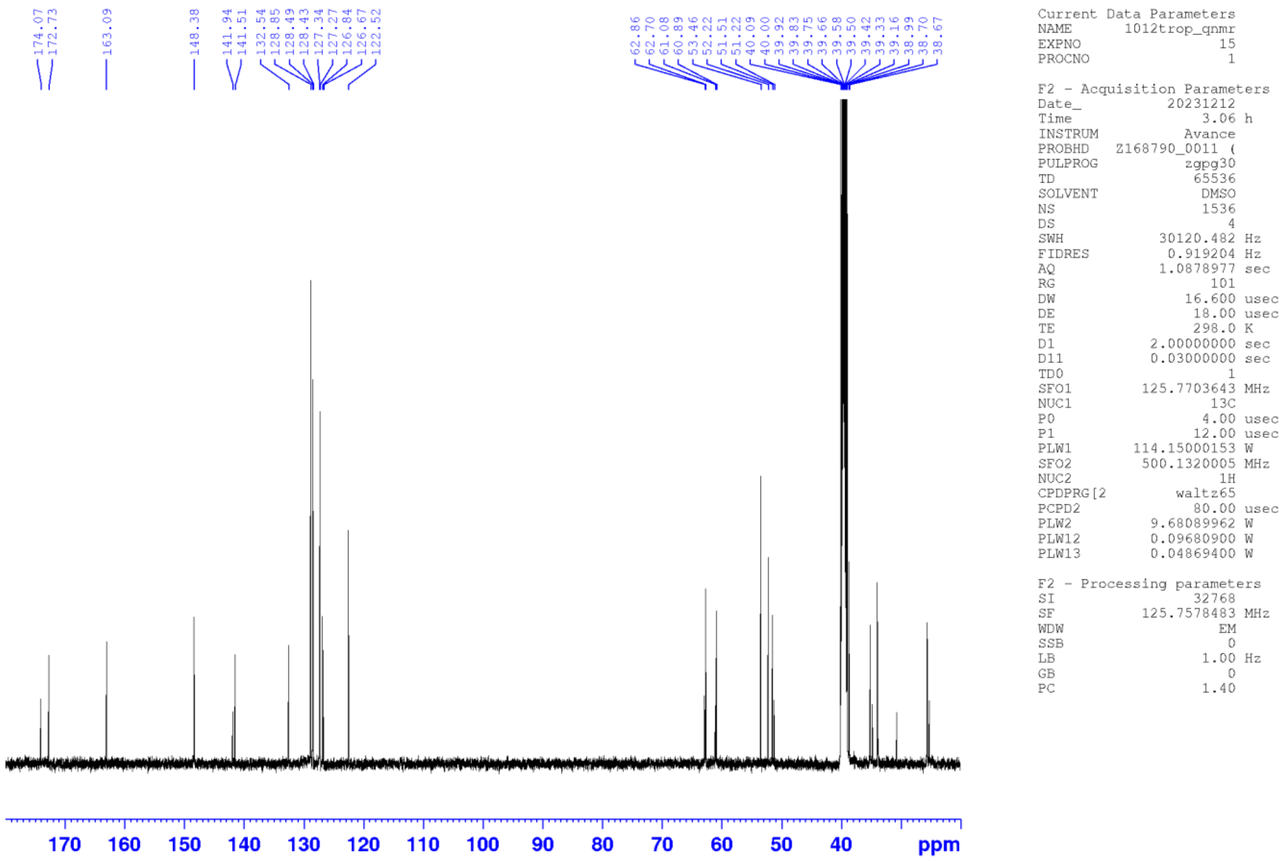

Figure S12 ^{13}C NMR spectrum of troparil sample.

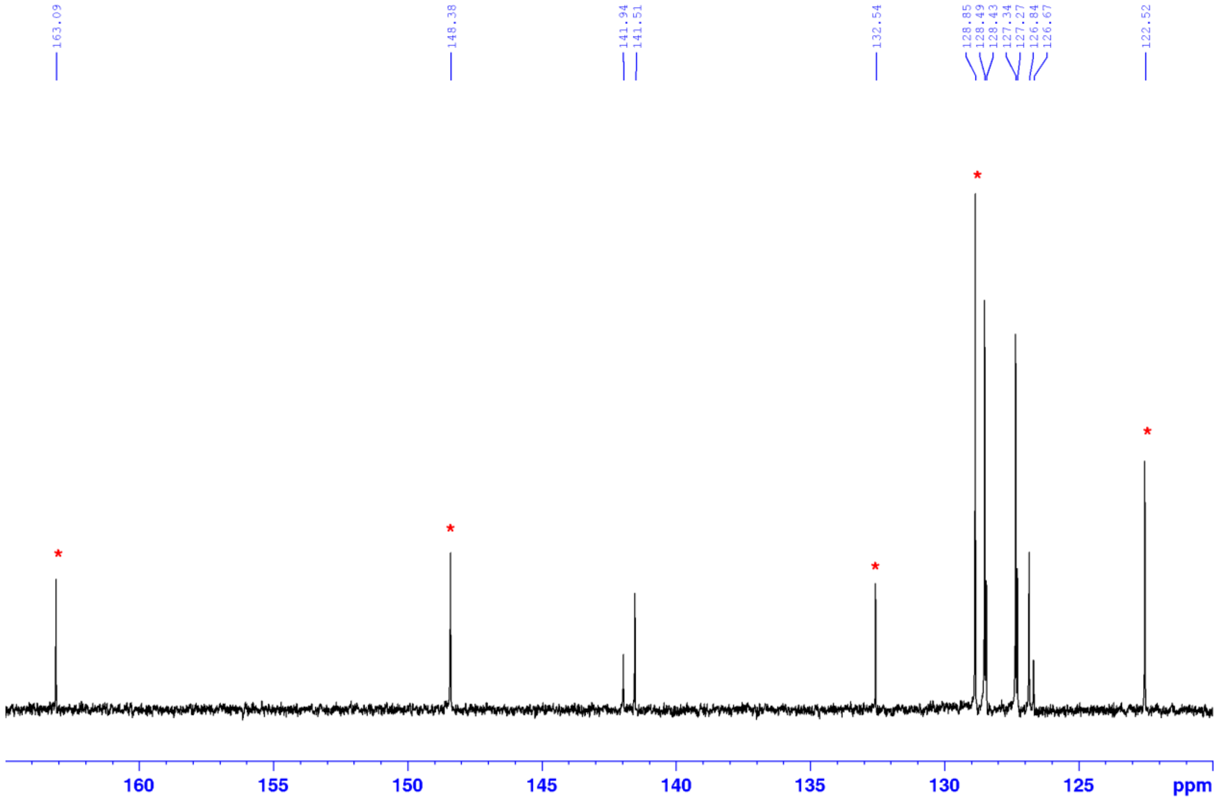

Figure S13 ^{13}C NMR spectrum of troparil sample, aromatic region. Signals marked with an asterisk originate from the internal qNMR standard methyl 3,5-dinitrobenzoate.

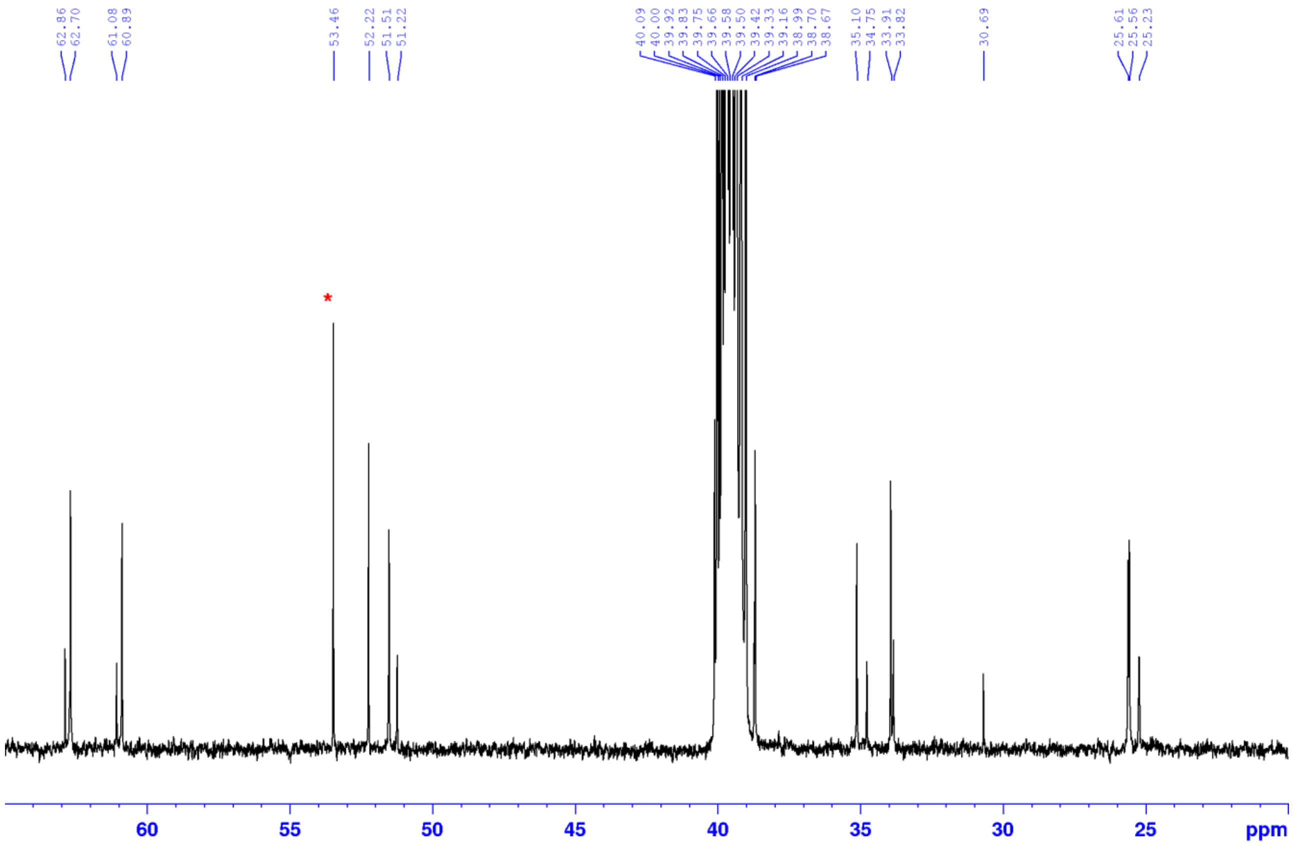

Figure S14 ^{13}C NMR spectrum of troparil sample, aliphatic region. Signal marked with an asterisk originate from the internal qNMR standard methyl 3,5-dinitrobenzoate.

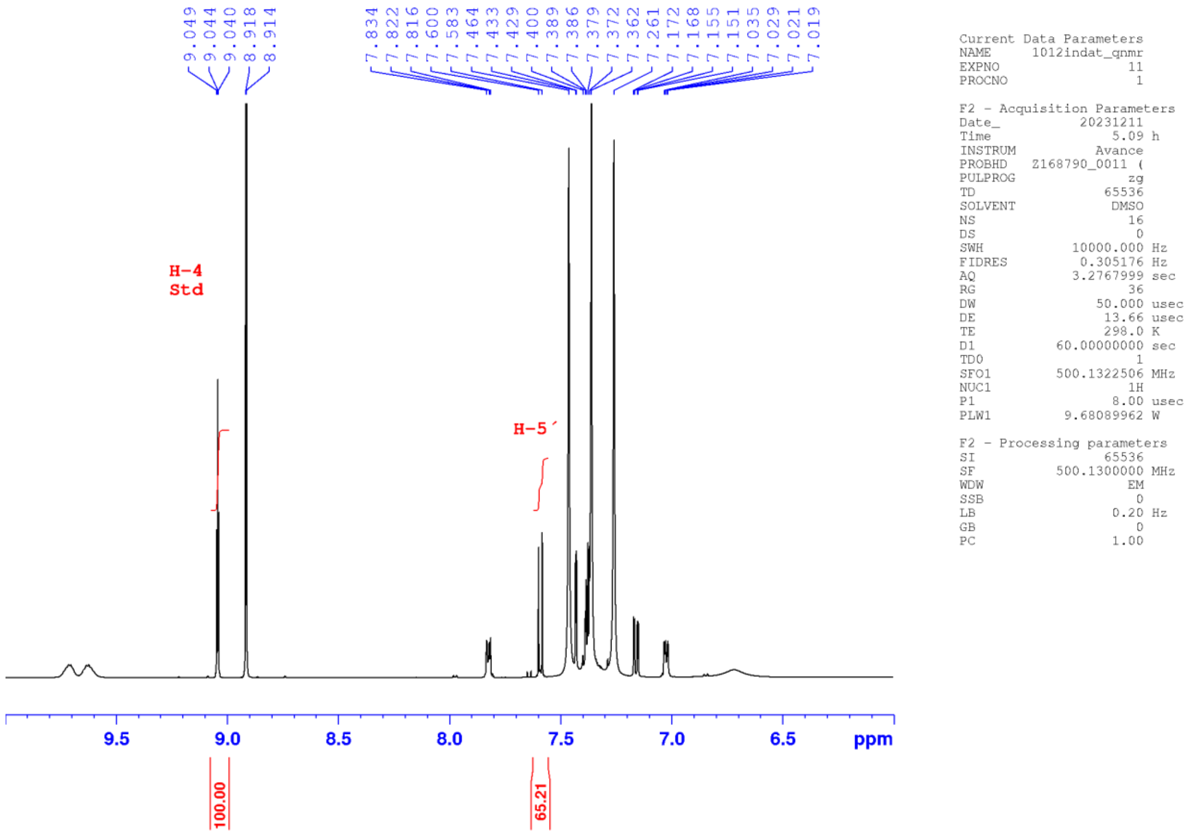

Figure S15 ^{1}H qNMR spectrum of indatraline sample, aromatic region, with integrals for the caclulation.

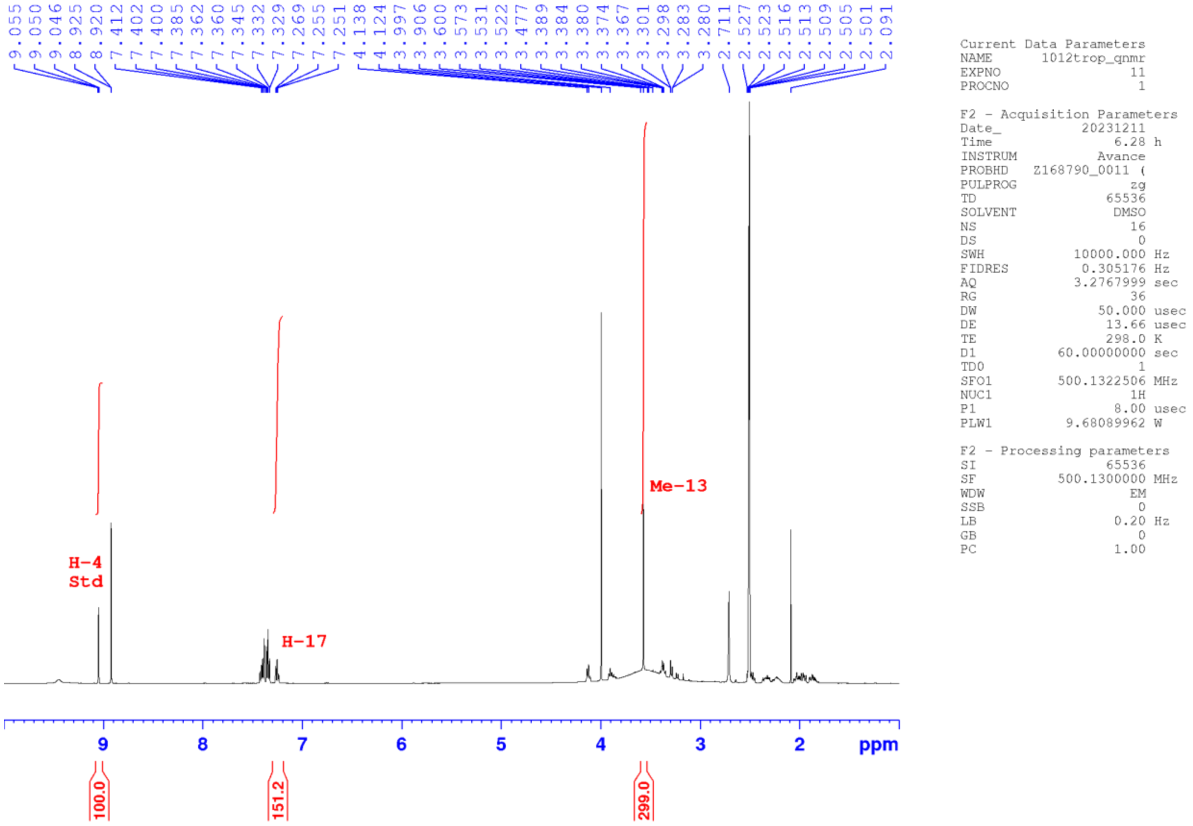

Figure S16 ^{1}H qNMR spectrum of troparil sample with integrals for the caclulations.

References

1 Songsoon Park and Hyeon-Kyu Lee. Efficient kinetic resolution in the asymmetric transfer hydrogenation of 3-aryl-indanones: applications to a short synthesis of (+)-indatraline and a formal synthesis of (R)-tolterodine. RSC Adv., 11, 23161, doi: 10.1039/D1RA04538E (2021).

2 Popławska M. et al. Identification and structural characterization of three psychoactive substances, phenylpiperazines (*p*BPP and 3,4-CFPP) and a cocaine analogue (troparil), in collected samples. Forensic Toxicology 40:132–143, doi: org/10.1007/s11419-021-00597-4 (2022)

3 Malz F. Quantitative NMR-[Spektroskopie als Referenzverfahren in der analytischen Chemie (PhD thesis)], Humboldt-Universität Berlin, Germany (2003)
